# A Zynq-Based Triaxial Vibration Sensing Station with GPS-Disciplined Timing

**DOI:** 10.3390/s26165089

**Published:** 2026-08-11

**Authors:** Xiyuan Zhang, Yongqing Wang, Qisheng Zhang, Mingwei Qi, Jinhang Zhang, Jingwen Zhang, Xiaochang Liu

**Affiliations:** Key Laboratory of Intraplate Volcanoes and Earthquakes, School of Geophysics and Information Technology, China University of Geosciences, Beijing 100083, China; 3010230015@email.cugb.edu.cn (X.Z.);

**Keywords:** vibration sensor system, triaxial acquisition, GPS-disciplined OCXO, time synchronization, embedded sensing, condition monitoring, Zynq MPSoC, deep drilling equipment

## Abstract

Deep drilling equipment operates under high-load, strong-vibration, intermittent-impact, and variable environmental conditions, which motivate sensing systems that provide low-noise acquisition, synchronized triaxial measurements, local data integrity, and quantitative measurement-chain characterization. This paper presents a Zynq UltraScale+ MPSoC-based triaxial vibration sensing station for deep drilling equipment applications. The modular station integrates conditioned-voltage triaxial accelerometer interfaces, analog signal conditioning, 24-bit simultaneous analog-to-digital conversion, electrical isolation, local solid-state-drive storage, Ethernet/wireless communication, and GPS-disciplined oven-controlled crystal oscillator (OCXO) timing. The programmable logic performs deterministic acquisition, GPS pulse processing, oscillator calibration, and DMA transfer, while the processing system facilitates storage, network communication, device-state management, and host computer interaction. The sensing electronics are evaluated through zero-input noise, an experiment-specific input-amplitude-to-noise ratio, gain linearity, thermal stability, repeatability, and station-to-station local-PPS timing tests. The characterized electronics achieve a mean equivalent input noise of 0.31 microvolts, a test-derived ratio of 135.08 dB, and a mean station-to-station local-PPS falling-edge difference of 0.34 microseconds. A lightweight post-acquisition interpretation workflow using learnable multichannel weighted fusion, a convolutional autoencoder, a training-distribution-based quantile threshold, and an auxiliary classification branch achieves 0.9705 accuracy and 0.9704 F1-score on a public triaxial bearing dataset under the reported protocol. A crane-based experiment evaluates deployment feasibility and the sensing–analysis workflow using controlled operating events and a removable stationary mass disturbance. The results provide an engineering sensing basis for distributed monitoring studies on deep drilling equipment.

## 1. Introduction

Deep scientific drilling provides direct access to information from the Earth’s crust and is an important tool for understanding deep geological structures, energy and mineral resources, and the mechanisms of major geohazards [1,2]. As drilling depth and operational complexity increase, drilling rigs are exposed to high loads, strong shocks, strong vibration, and coupled mechanical and environmental disturbances. Drillstring dynamics studies have shown that stick–slip, whirl, and coupled vibration modes can strongly affect drilling performance and tool life [3,4]. Potential monitoring targets in such systems include rotating, hoisting, transmission, and load-bearing assemblies, where wear, fatigue damage, misalignment, looseness, impact, and abnormal vibration may occur. Degradation or failure of these assemblies can reduce operational efficiency, increase maintenance cost, and introduce safety risks. Reliable online condition monitoring and early fault warning are consequently important for the safe and efficient operation of deep drilling equipment.

Vibration signals are among the most sensitive indicators of the health of rotating machinery and transmission systems [5,6]. Compared with temperature, pressure, or current measurements, vibration measurements often reveal incipient bearing defects, structural looseness, imbalance, and misalignment at an earlier stage. Vibration-based condition monitoring has therefore become a central technique in machinery diagnostics and prognostics [7,8]. At the same time, advances in high-performance sensors, embedded computing, industrial networks, and edge computing have promoted distributed acquisition systems for complex industrial environments [9,10,11,12,13]. Deep learning has also improved automated feature extraction, fault recognition, and anomaly detection from raw vibration signals [14,15,16,17]. More recent Transformer and self-supervised approaches improve long-range representation learning and reduce the dependence on labelled fault data, respectively, but their data and computation requirements remain important deployment considerations [18,19,20].

However, several challenges remain when vibration monitoring is transferred from laboratory machinery to deep drilling applications. First, drilling sites exhibit strong background noise, multiple excitation sources, rapidly changing operating conditions, and limited network availability. These characteristics make many laboratory-oriented data acquisition and diagnostic schemes difficult to deploy directly in the field. Vibration signature analysis has been used for drilling tool condition monitoring, but transferring such signal-based diagnosis to rig-scale field equipment requires stable acquisition, synchronization, and robust processing [21]. Second, measurement quality is often treated as a secondary implementation detail, although field vibration records are strongly affected by the noise floor, analog front-end gain error, channel-to-channel timing consistency, oscillator stability, storage continuity, and environmental drift. Commercial instruments provide mature acquisition functions, but a custom station allows the sensor interface, local high-capacity storage, GPS-disciplined timing, and PL-level deterministic control to be integrated and characterized for this specific deployment study. Third, edge and cloud systems improve connectivity and scalable processing, but intermittent field links make local storage and autonomous acquisition necessary [12,13]. Finally, manually fixed anomaly thresholds are sensitive to changes in speed, load, and structural response, while simple equal-weight fusion neglects the different sensitivities of individual axes or sensor locations [22,23,24,25].

From a sensing-system perspective, the key objective is to improve the full sensor-to-decision chain, including synchronized signal acquisition, edge-side data integrity, time-referenced distributed measurement, and quantitative performance characterization. For a monitoring system intended for field deployment, diagnosis accuracy alone is insufficient unless the measured signals can be related to a defined acquisition bandwidth, noise floor, gain-linearity response, thermal stability, and timing reference. Therefore, this work treats the acquisition station as an integrated vibration sensing instrument first and the diagnostic workflow as a post-acquisition interpretation layer built on top of that instrument.

To address these issues, this paper develops a Zynq-based triaxial vibration sensing and condition-monitoring station for deep drilling rig applications. The hardware integrates multichannel signal conditioning, 24-bit simultaneous sampling, electrical isolation, local mass storage, wired and wireless communication, and GPS + OCXO time synchronization. The software exploits the heterogeneous processing capability of the Zynq MPSoC by assigning deterministic acquisition and timing tasks to the programmable logic (PL) and system management, storage, transmission, and human–machine interaction tasks to the processing system (PS). At the interpretation level, a learnable multichannel weighted fusion strategy is combined with a training-distribution-based quantile autoencoder and a lightweight convolutional classification branch to provide both binary health-state judgement and multiclass state recognition from acquired vibration records.

The main contributions of this study, with emphasis on the sensing-system design and validation, are as follows:

1. A modular Zynq-based triaxial vibration sensing station is developed for field-oriented deep drilling applications, integrating low-noise acquisition, PL-side deterministic control, PS-side data management, local storage, and wired/wireless communication.
2. A GPS + OCXO clock-calibration method is implemented in the FPGA, combining multi-PPS gate frequency measurement and DAC-based oscillator control to discipline the local OCXO and support distributed synchronized acquisition.
3. The sensor-to-decision measurement chain is quantitatively characterized through noise, dynamic-range, gain-linearity, thermal-stability, repeatability, and station-to-station synchronization tests.
4. A post-acquisition condition-monitoring workflow based on learnable multichannel weighted fusion and quantile-threshold anomaly detection is evaluated using a public triaxial bearing dataset and a crane-based rig analogue field test.

## 2. System Overview

The proposed system is designed for long-term vibration monitoring in deep drilling rig application scenarios, where network access, manual maintenance, and operating conditions can be unstable. It therefore combines local acquisition and storage with real-time transmission and post-acquisition analysis. The acquisition station receives triaxial acceleration signals, performs analog conditioning and analog-to-digital conversion, stores data locally, transmits selected data streams to a host computer, and provides synchronized timing for distributed deployment.

**Figure 1:**
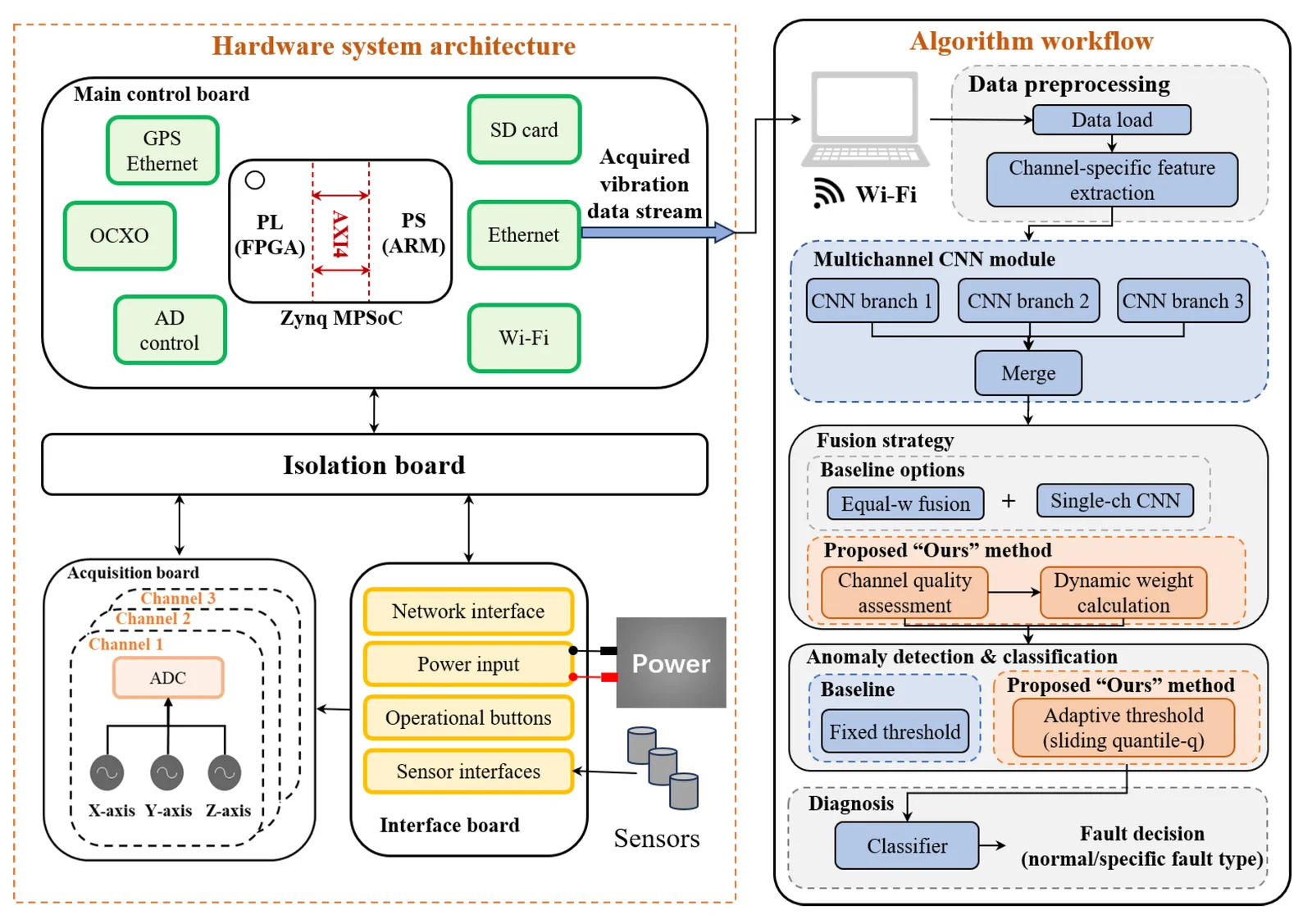
Overall architecture of the vibration acquisition and diagnosis system. The left part shows the hardware acquisition station, and the right part shows the post-acquisition diagnosis workflow.

The overall architecture is shown in Figure 1. The acquisition station consists of four main hardware modules: an acquisition board, a main control board, an isolation board, and an interface board. The station is powered by a 24 V supply from the rig. The acquisition board contains three triaxial sensor ports, corresponding to nine analog measurement axes, and performs filtering, gain adjustment, single-ended-to-differential conversion, and analog-to-digital conversion. The main control board is built around a Zynq UltraScale+ MPSoC and is responsible for data aggregation, acquisition control, time synchronization, local storage, and network communication. The isolation board suppresses electrical interference between the analog front end and the digital control board. The interface board provides power management, sensor interfaces, operation buttons, network connectors, and status-related connections.

**Figure 2:**
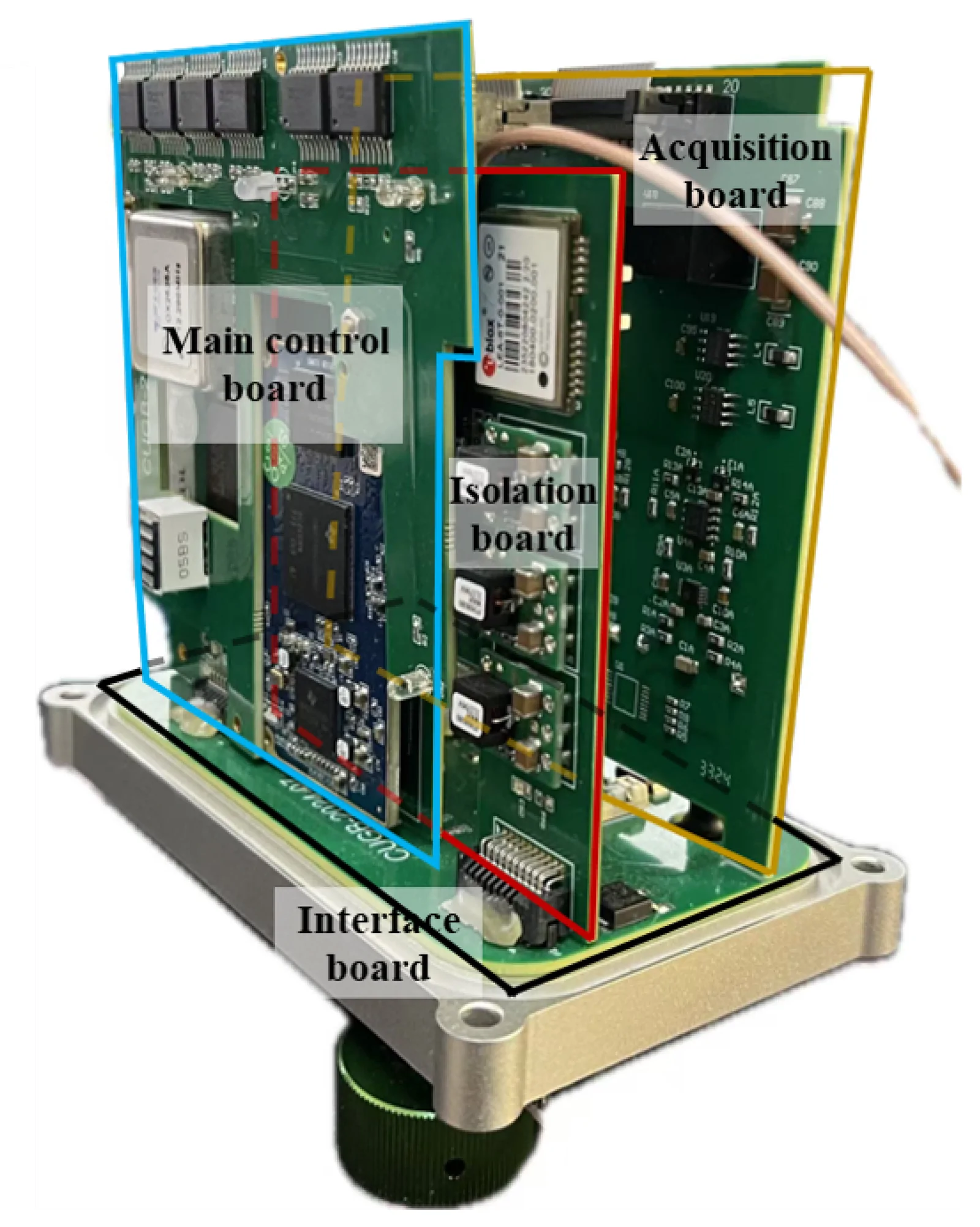
Hardware assembly of the acquisition station, including the main control board, acquisition board, isolation board, and interface board.

The mechanical integration of the four boards is shown in Figure 2. The interface board is mounted on the side of the enclosure, while the main control board, isolation board, and acquisition board are connected to it through board-to-board connectors. This layout improves integration density and allows the analog front end, digital control unit, and external interfaces to be maintained or upgraded separately.

## 3. Key Technologies

### 3.1. Multi-Channel Acquisition Circuit

**Figure 3:**
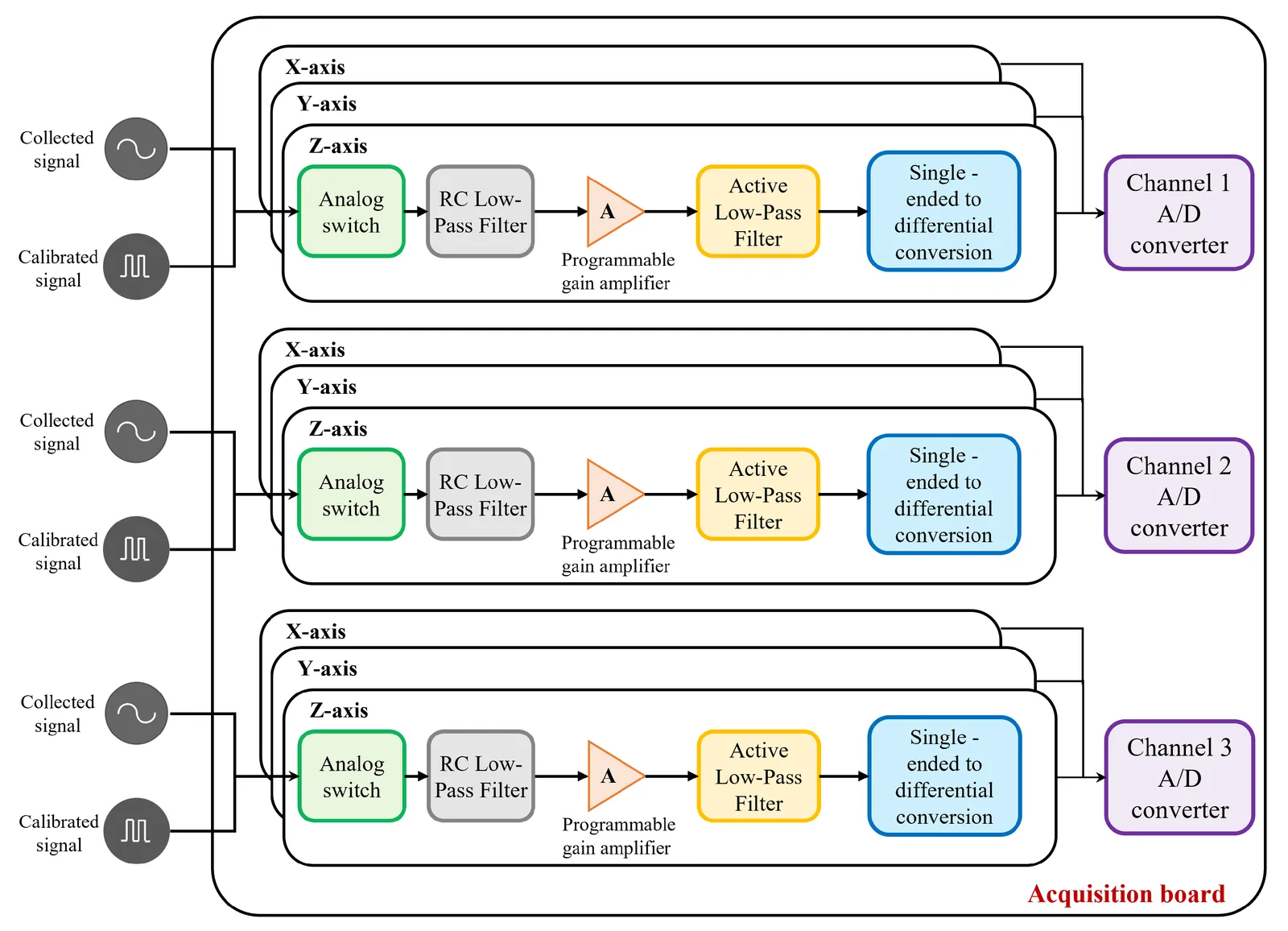
Signal-conditioning and acquisition circuit of the acquisition board. Each channel supports triaxial acceleration input, analog filtering, programmable gain, differential conversion, and 24-bit ADC sampling.

The acquisition board is designed to acquire three triaxial acceleration signals, corresponding to nine vibration axes in total. Its circuit structure is shown in Figure 3. Each axis first passes through an analog switch and an RC low-pass filter. The signal is then amplified by a programmable-gain instrumentation amplifier and further filtered by a second-order active low-pass filter. Finally, the single-ended signal is converted to a differential signal that meets the input requirements of the analog-to-digital converter.

The converter used in the system is the ADS1274 (Texas Instruments, Dallas, TX, USA), a 24-bit, four-channel, simultaneous-sampling delta-sigma ADC [26]. Each station contains three ADS1274 devices. Each converter digitizes the three axes of one triaxial sensor, with its fourth input unused; all three converters share the sampling clock and control signals. The use of simultaneous sampling avoids phase differences introduced by multiplexed acquisition and is suitable for triaxial vibration analysis. The converted data are transferred through the isolation board to the Zynq device for subsequent processing. The acquisition mode and calibration mode can be configured by software, enabling the same hardware to support normal measurement, zero-input noise tests, and calibration tasks.

The ADS1274 output sampling rate is adjusted by changing the master clock supplied by the FPGA. Its internal linear-phase FIR/decimation filter operates along the normal conversion path, with a passband extending to $0.453f_{\mathrm{DATA}}$ and a stopband beginning at $0.547f_{\mathrm{DATA}}$ [26]. The fixed 10 kHz analog low-pass filter limits high-frequency content entering the converter, while the ADS1274 internal filter provides sampling-rate-dependent digital filtering. At lower sampling rates, cascaded FIR filters in the FPGA are additionally enabled to further limit the signal bandwidth and suppress out-of-band components without changing the sampling rate. The resulting accelerometer and acquisition-interface specifications are summarized in Table 1.

### 3.2. Zynq-Based Real-Time Data Processing

#### 3.2.1. Main Control Board Hardware

**Figure 4:**
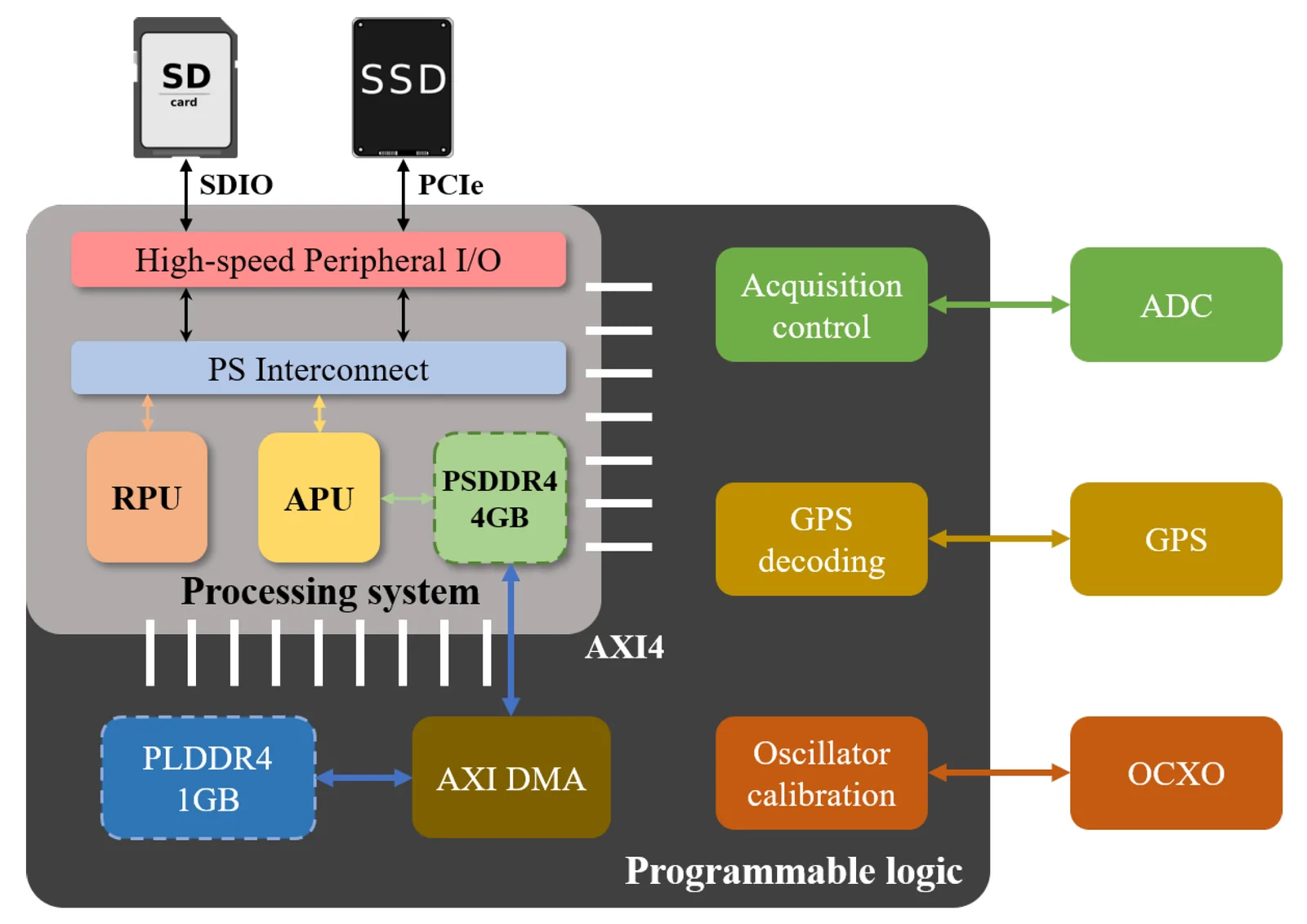
Zynq MPSoC hardware architecture of the main control board. The PS handles system-level tasks, while the PL performs acquisition control, GPS decoding, oscillator calibration, and DMA-based data transfer.

The main control board is designed around a core-board-plus-custom-digital-board structure. The PS/PL partition and associated interfaces are shown in Figure 4. The core board uses a Xilinx Zynq UltraScale+ XCZU3EG MPSoC (Advanced Micro Devices, Inc., Santa Clara, CA, USA), which integrates an Arm-based PS and FPGA-based PL on the same device [27,28]. Such reconfigurable MPSoC architectures are well suited to applications that require both software flexibility and deterministic low-level processing [29]. The PS contains Arm Cortex-A53 and Cortex-R5 processors and is suitable for embedded Linux, system-state management, data scheduling, communication control, and human–computer interaction. The PL provides parallel logic resources, on-chip memory, and DSP resources and is used for deterministic acquisition control, low-level interface management, GPS pulse processing, oscillator calibration, and high-throughput data transfer.

The board expands storage, communication, timing, debugging, and power-management resources according to the requirements of field deployment. A PCIe M.2 interface is used to connect a high-speed solid-state drive for long-duration vibration data storage. A Gigabit Ethernet PHY and RJ45 connector provide stable wired communication with the host computer. Wireless communication is also reserved for situations where cable deployment is inconvenient. A microSD interface supports system boot and removable storage, and a UART interface supports low-level debugging and maintenance.

GPS-disciplined time synchronization is implemented using a GPS receiver, an oven-controlled crystal oscillator (OCXO), and a digital-to-analog converter (DAC) calibration circuit. The GPS module provides an external pulse-per-second (PPS) reference. The main control board captures the PPS signal and uses it to calibrate the local OCXO. Compared with relying only on discrete GPS pulses, the GPS + OCXO scheme combines long-term time accuracy with short-term frequency stability, which is important for continuous acquisition and distributed multi-station deployment [30,31].

#### 3.2.2. Time Synchronization and Clock Calibration

**Figure 5:**
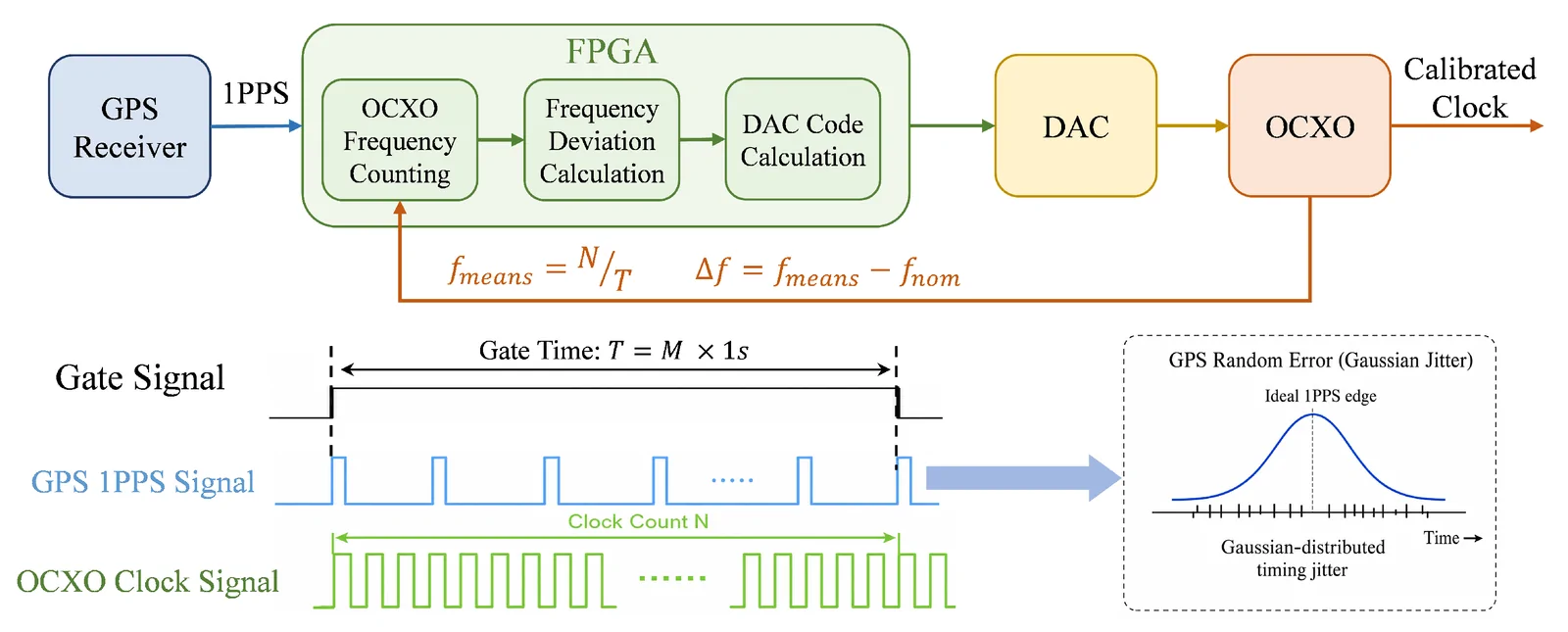
GPS + OCXO time-synchronization and clock-calibration architecture. The FPGA measures the OCXO frequency against the GPS PPS reference, controls the DAC voltage to discipline the OCXO, generates divided reference signals for the analog-board calibration circuit, and updates system time using UTC information parsed from NMEA-0183 messages.

The time-synchronization architecture is shown in Figure 5. The acquisition-station clock system consists of the FPGA, a LEA-M8S-0-10 GPS receiver with a BT-6020 antenna, an OX2525A-12.288 MHz OCXO, and a DAC. The GPS receiver provides a PPS reference and NMEA-0183 serial messages, while the OCXO provides the local high-stability clock used by the acquisition station. The FPGA receives the GPS PPS signal, measures the OCXO frequency, calculates the corresponding frequency deviation, and outputs a DAC control quantity to tune the OCXO frequency.

The oscillator-calibration module contains two parts: a frequency-measurement submodule and a DAC-control submodule. The frequency-measurement submodule receives the OCXO clock and the GPS PPS reference, compares the local oscillator count with the nominal reference interval, and estimates the frequency deviation between the OCXO and the standard frequency. The DAC-control submodule then generates the corresponding analog control voltage for the OCXO. Through iterative measurement and voltage adjustment, the OCXO output frequency is gradually pulled toward the GPS-disciplined reference.

Because the GPS PPS contains random timing error, using only one PPS interval as the calibration gate would transfer short-term GPS jitter directly into the oscillator-control loop. Therefore, the calibration gate is formed from the accumulated interval of multiple PPS pulses. This longer gate time averages the random PPS error and improves the stability of the estimated OCXO frequency deviation.

After oscillator calibration, the signal-calibration module receives the disciplined local clock and divides it to generate square-wave reference signals with configurable frequencies. The division coefficient is configured by the command-control module, and the generated reference signal is output to the calibration circuit on the analog board. This function allows the acquisition chain to be checked using a locally generated timing-related reference signal.

The real-time-clock module parses NMEA-0183 messages to obtain UTC time and geographic coordinates. The GPS PPS edge is used for hardware-level time alignment, and the parsed UTC information is used to initialize and update the system time. The longitude and latitude information is uploaded to the command-control module for station-state reporting and field deployment records. The station declares timing lock only when GPS timing is valid, the OCXO has completed warm-up, and the PPS count error remains below two local-clock periods for ten consecutive comparisons. If GPS is lost, the FPGA holds the last DAC setting, continues timing from the OCXO, flags the loss, and immediately attempts resynchronization when GPS becomes available.

#### 3.2.3. Embedded Software

The embedded software follows the PL/PS task partition of the Zynq MPSoC. The PL implements ADC timing, GPS/PPS capture, OCXO frequency calibration, low-level interface control, and AXI-DMA data transfer. The PS runs embedded Linux to manage SSD storage, wired/wireless communication, device-state monitoring, host commands, and data packaging for host-side analysis. This division keeps acquisition and timing deterministic while leaving file management, networking, and user interaction to the software.

### 3.3. Intelligent Condition-Monitoring Method

#### 3.3.1. Overall Framework

**Figure 6:**
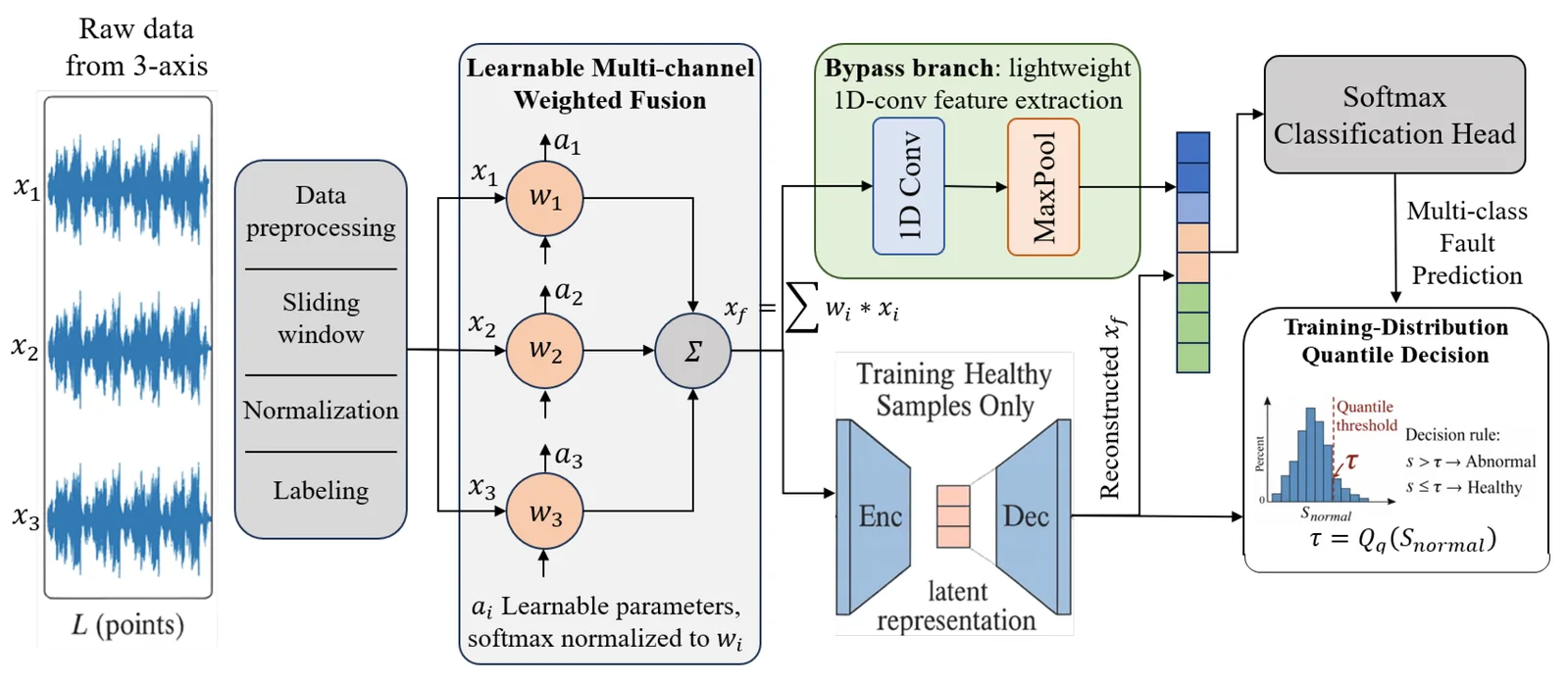
Proposed condition-monitoring method based on learnable multichannel weighted fusion, convolutional autoencoding, training-distribution-based quantile judgement, and an auxiliary classification branch.

After the hardware acquisition platform is established, an intelligent condition-monitoring method is developed for the triaxial vibration data generated by the acquisition station. Drilling sites contain multiple excitation sources, strong noise, and variable operating conditions. Traditional single-channel analysis and manually fixed thresholds are therefore often insufficient for robust field monitoring [24,32,33]. The proposed method, shown in Figure 6, consists of five parts: data preprocessing, multichannel feature extraction, learnable weighted fusion, one-dimensional convolutional autoencoding with training-distribution-based quantile detection, and multiclass state classification.

The algorithmic design is a lightweight host-side interpretation layer for the proposed measurement station. Its components and comparison baselines are related to established research families, including support vector machines for statistical classification [34], deep CNN and one-dimensional CNN methods for vibration-based fault diagnosis [14,16,17,35], autoencoder reconstruction-error methods for anomaly detection [25], LSTM prediction-error and thresholding methods [32,33], and multi-sensor fusion or attention-based methods for multichannel mechanical diagnosis [22,36,37,38]. This literature context is used to define representative baseline implementations and ablation models under the same experimental settings.

Raw vibration signals are first segmented by a sliding window, normalized, and labelled. The three synchronized input channels are then processed by a learnable channel-weighting module that produces a single fused sequence. A convolutional autoencoder learns a compact latent representation and reconstructs the fused signal. The reconstruction error is used as an anomaly score. In parallel, a lightweight convolutional bypass branch extracts discriminative features from the original three-channel input so that channel-specific differences are not lost during fusion. The latent representation and bypass features are concatenated and passed to a softmax classification head for multi-class state recognition.

#### 3.3.2. Learnable Multi-Channel Weighted Fusion

For a window of aligned triaxial vibration signals, the three input sequences are denoted by

(1) $$x_{1},x_{2},x_{3}\in R^{L},$$

where *L* is the window length. To reflect the different fault sensitivities of different axes without greatly increasing model complexity, each channel is assigned a trainable parameter $a_{i}$. A softmax function converts these parameters into normalized channel weights:

(2) $$w_{i}=\frac{\exp(a_{i})}{\sum_{j=1}^{3}\exp\left(a_{j}\right)},\;i=1,2,3,$$

where $w_{i}\geq 0$ and $\sum_{i=1}^{3}w_{i}=1$. The fused sequence is then

(3) $$x_{f}=\sum_{i=1}^{3}w_{i}x_{i},$$

where $x_{f}\in R^{L}$. During end-to-end training, the channel weights are updated together with the reconstruction and classification objectives. The model can therefore increase the contribution of fault-sensitive channels and suppress noisy or redundant channels. Compared with fixed equal-weight averaging, this mechanism better reflects the anisotropic vibration responses commonly observed in mechanical structures.

#### 3.3.3. Training-Distribution-Based Quantile Anomaly Detection

The reconstruction objective of the convolutional autoencoder is trained using healthy samples, whereas the auxiliary multiclass classification objective uses all labelled training samples. Let the anomaly-score set of healthy training samples be

(4) $$S_{\mathrm{normal}}=\left\{s_{1},s_{2},\ldots ,s_{N}\right\},$$

where $s_{i}$ is the reconstruction error of the *i*th healthy window. For a selected quantile level *q*, the anomaly threshold is

(5) $$\tau =Q_{q}\left(S_{\mathrm{normal}}\right).$$

A test sample is classified as abnormal if

(6) s>τ,

and healthy otherwise. The threshold is estimated once from the healthy training distribution and remains fixed during validation and testing; therefore, “adaptive” here refers to data-derived calibration rather than online updating. Online updating of the threshold under long-duration distribution shift remains a topic for future work.

#### 3.3.4. Benchmark Experiment

The proposed method is first evaluated on the Triaxial Bearing Vibration Dataset of an induction motor under varying load conditions [39,40]. This dataset is selected because its triaxial vibration records match the triaxial input structure of the proposed acquisition station. It contains triaxial vibration signals collected from bearings under different loads, fault types, and fault sizes at a sampling rate of 10 kHz. The original signals are segmented using a window length of 1024 samples and a step size of 512 samples.

To avoid leakage between overlapping windows, the dataset is first stratified by class and divided at the level of complete original recordings into training, validation, and test subsets, with a target ratio of 70:15:15. Each original recording is assigned to only one subset. Windowing is then performed separately within each subset using a window length of 1024 samples and a step size of 512 samples. Consequently, overlapping windows from the same recording cannot appear in both the training and test sets. Normalization parameters and the training-distribution-based quantile threshold are estimated only from normal samples in the training set. The training set is used for parameter learning, the validation set is used for hyperparameter tuning and model selection, and the test set is used for final evaluation. To reduce the effect of random initialization and sampling, the experiments are repeated 10 times under the same settings, and the mean results are reported. Two types of comparisons are conducted. First, the proposed method is compared with representative baseline implementations, including SVM, 1D-CNN, WDCNN, and AE with thresholding. Second, ablation variants are evaluated to isolate the contributions of weighted fusion, reconstruction-based anomaly detection, and the auxiliary classification branch. All compared methods are implemented using the same recording-level split, window length, sample number, and evaluation protocol.

The encoder contains two one-dimensional convolutional blocks with 48 and 96 channels (kernel size 15, stride 4) followed by a 64-dimensional latent layer. A two-layer triaxial bypass with 48 and 64 channels is globally pooled and concatenated with the latent vector; the classifier uses hidden dimensions of 192 and 96. The weighted training objective uses reconstruction and classification coefficients of 0.75 and 2.4, respectively, with 0.04 label smoothing. Each class contains 500 samples in the reported comparison. The model uses 964,040 trainable parameters and is trained for 60 epochs with AdamW (learning rate 0.0008, weight decay 0.0001, and batch size 48). Reconstruction loss is evaluated on healthy training windows, while classification loss is evaluated on all labelled training windows. In this study, trained models were run on the host computer after acquisition rather than on the Zynq processing system. On an Intel Core i7-8700 (Intel Corporation, Santa Clara, CA, USA) using six CPU threads and batch size 1, the complete in-memory decision path has a mean latency of 2.681 ms, a P95 latency of 3.269 ms, and a peak RSS increment of 21.77 MiB. These figures exclude sensor acquisition, disk/network I/O, and application queueing. AE denotes autoencoder. As shown in Table 2, the proposed method achieves the highest reported mean accuracy, precision, recall, and F1-score under this experimental protocol. The 1D-CNN baseline is the strongest comparison method, with an accuracy of 0.9685 and an F1-score of 0.9685, whereas the proposed method obtains 0.9705 and 0.9704, respectively. The ablation results in Table 3 indicate the contributions of the weighted-fusion and auxiliary-branch design. The fixed quantile-threshold branch supports binary health/abnormal judgement but is not updated during testing.

## 4. Tests and Results

### 4.1. Hardware Performance Test

The background noise and dynamic range of the acquisition station are tested at a sampling rate of 1 kHz and a gain of 1. In the background-noise test, the differential input of the acquisition channel is shorted and data are acquired without external excitation. The equivalent input noise is calculated from the sampled voltage sequence as follows:

(7) $$V_{\mathrm{noise}}=\frac{\sqrt{\frac{1}{N}\sum_{i=1}^{N}{\left(V_{i}-\bar{V}\right)}^{2}}}{G},$$

where $V_{i}$ is the *i*th sampled voltage, $\bar{V}$ is the mean voltage, *N* is the sample number, and *G* is the gain. Ten repeated measurements give a mean equivalent input noise of 0.31 μV, a standard deviation of 0.008 μV, a minimum of 0.29 μV, and a maximum of 0.33 μV, characterizing the low-noise performance of the acquisition channel.

In the dynamic-range test, a sinusoidal excitation from a signal generator is applied to the input. The amplitude is gradually increased until visible saturation distortion appears in the output waveform. The maximum unsaturated RMS input voltage is approximately 1.767 V. The dynamic range is calculated as follows:

(8) $$\mathrm{DR}=20\log_{10}\left(\frac{V_{\max}}{V_{\mathrm{noise}}}\right),$$

which gives a mean value of about 135.08 dB in the repeated test. This is an experiment-specific ratio between the measured maximum unsaturated RMS input and the shorted-input RMS noise under the stated 1 kHz, gain-1 conditions. It is not a manufacturer-standard dBFS dynamic-range specification and therefore cannot be compared directly with the commercial dBFS values in Table 4.

The self-developed station is also compared functionally with two commercial vibration acquisition devices, the Dewesoft IOLITE-8xACC and the NI-9232, using public manufacturer specifications [41,42]. The comparison in Table 4 summarizes the channel configuration, resolution, synchronization, storage, and communication functions most relevant to field monitoring.

### 4.2. Calibration and Linearity Verification

Calibration and linearity verification were carried out using short-input offset, multi-level sine-input, and warm-up tests. The short-input offset is the DC mean value of the sampled sequence after the input is shorted, whereas the equivalent input noise reported in Section 4.1 is the RMS fluctuation around that mean.

The gain error at test point *i* is

(9) $$e_{g,i}=\frac{V_{\mathrm{meas},i}-V_{\mathrm{ref},i}}{V_{\mathrm{ref},i}}\times 100\%,$$

and the offset error is

(10) $$e_{\mathrm{off}}=V_{\mathrm{meas},0}-V_{\mathrm{ref},0}.$$

For gain and linearity verification, a 50 Hz sinusoidal reference was applied with 8 amplitude levels from 10 mV_pp_ to 5000 mV_pp_ at gain 1. The theoretical RMS reference was computed by $V_{\mathrm{rms}}=V_{\mathrm{pp}}/\left(2\sqrt{2}\right)$. Linear fitting between measured and reference RMS values gives

(11) $$V_{\mathrm{meas}}=1.000031\;V_{\mathrm{ref}}+0.236\;\mathrm{mV},$$

with $R^{2}=0.99999937$. The maximum fitting residual is 0.935 mV (≈0.099% FS, where FS is the 5000 mV_pp_ point), the mean relative gain error is −0.080%, and the maximum absolute relative error is 1.288% at the 10 mV_pp_ low-amplitude point. These results are summarized in Table 5.

Thermal stability was evaluated at room temperature using a 50 Hz, 1000 mV_pp_ sinusoidal input, a sampling rate of 1 kHz, and gain 1. The equipment temperature and measured RMS voltage were recorded from cold start to 240 min, as shown in Table 6. The theoretical RMS value of the input signal is 353.553 mV. During the 240 min test, the measured RMS value varied from 354.22 mV to 354.57 mV, corresponding to a drift range of 0.35 mV. The gain error decreased from 0.288% at startup to about 0.189% after 240 min, with a total gain-error range of 0.099 percentage points. After approximately 30 min, the measured RMS response stabilized within 354.22–354.25 mV.

### 4.3. Repeatability Evaluation

For repeated runs under identical settings, repeatability is quantified by the coefficient of variation:

(12) $$\mathrm{CV}=\frac{\sigma_{x}}{\mu_{x}}\times 100\%,$$

where $\mu_{x}$ and $\sigma_{x}$ are the mean and standard deviation of repeated measurements. For channel-8 offset under short-input condition, 10 repeated runs give a DC mean of 592 nV, a sample standard deviation of 22.5 nV, and a drift range of 550–620 nV. The results are summarized in Table 7.

### 4.4. Long-Duration Storage Test

Continuous local storage was tested for 72 h using the station’s binary-file format. The software write counter accumulated 135,895,384,064 bytes, and the summed file sizes matched this counter. The recorded DMA, read, write, and process error counters all remained zero. Because the binary files include framing and alignment padding, the total byte count is not interpreted as a pure 27-byte sampling payload. This test evaluates local-storage continuity.

### 4.5. Synchronization Accuracy Evaluation

**Figure 7:**
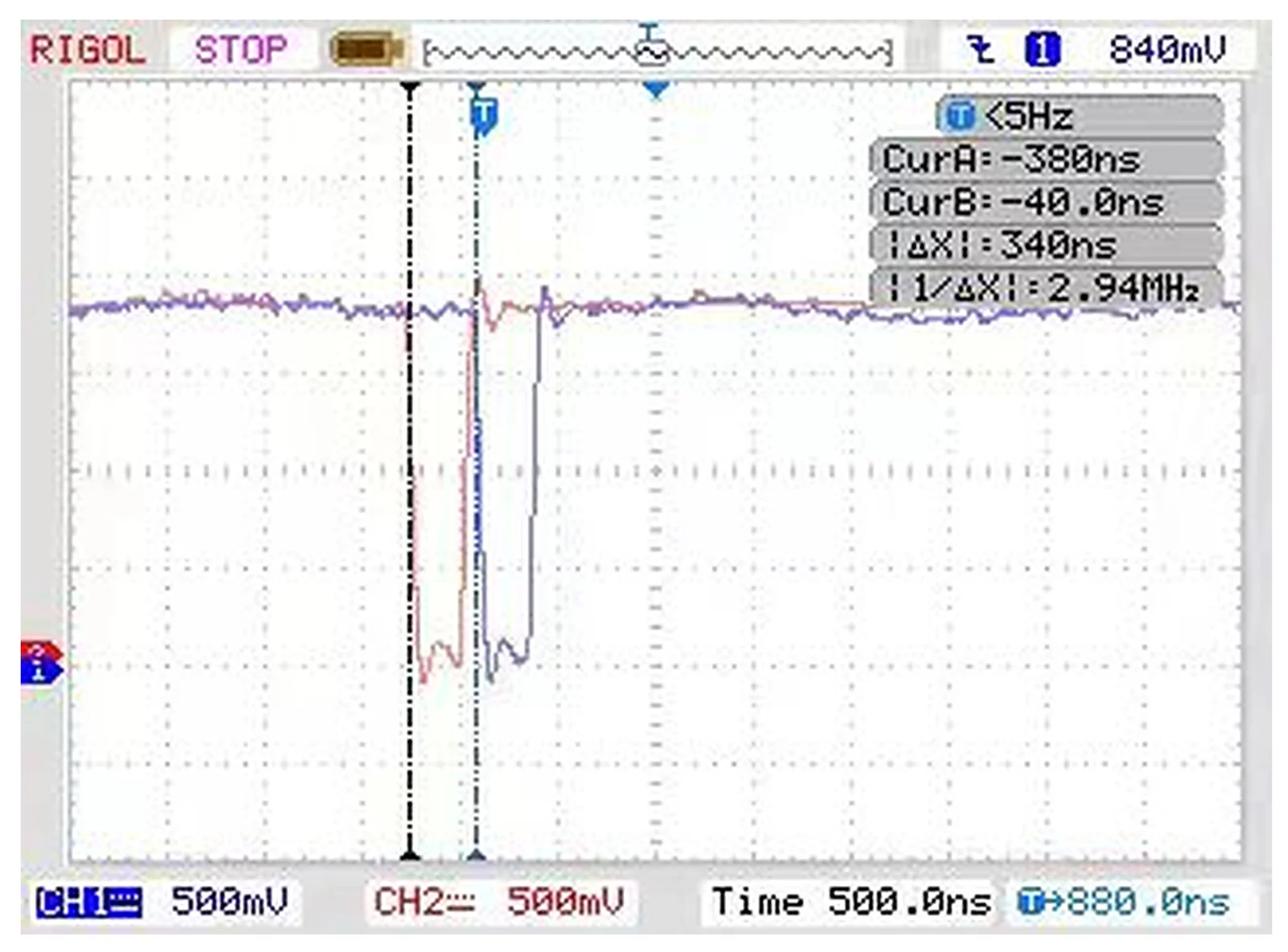
Station-to-station synchronization test using the locked local pulse-per-second outputs of two acquisition stations. The GPS receiver and the OX2525A-12.288 MHz OCXO are locked before the falling-edge time difference is measured using an oscilloscope.

Station-to-station timing is first evaluated by a pulse-edge method, as shown in Figure 7. After the GPS receiver and the OX2525A-12.288 MHz OCXO are locked, the local pulse-per-second outputs of two acquisition stations are measured by an oscilloscope. Because the local PPS signal remains high between pulses, the falling edge is used as the timing reference, and the falling-edge time difference is recorded. Ten repeated measurements give a mean station-to-station edge difference of 0.34 μs, a standard deviation of about 0.04 μs, a minimum of 0.32 μs, and a maximum of 0.38 μs. This pulse-edge result characterizes consistency between the two local PPS outputs; recorded-signal synchronization across the acquisition paths is evaluated separately below.

**Figure 8:**
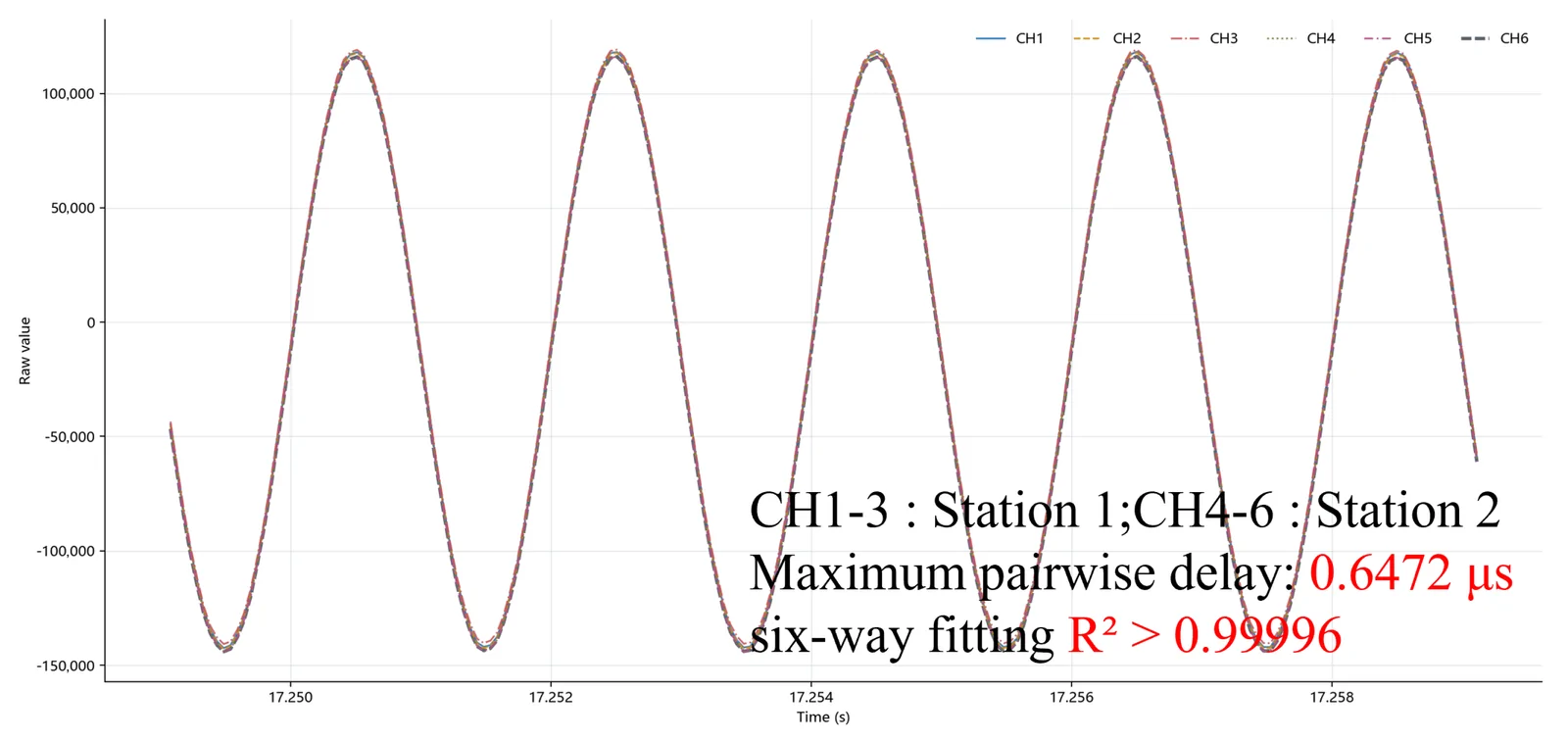
Recorded-signal synchronization result for two acquisition stations. CH1–CH3 are channels from Station 1 and CH4–CH6 are channels from Station 2. Sinusoidal fitting gives a maximum pairwise delay of 0.6472 μs with $R^{2}>0.99996$.

A recorded-signal comparison is shown in Figure 8. The local PPS results are summarized in Table 8. Three channels from each of two stations recorded the same periodic input. Sinusoidal fitting gives a maximum pairwise delay of 0.6472 μs across the six channels, with $R^{2}>0.99996$. This result includes the acquisition paths and therefore complements the local-PPS edge test.

GPS-loss holdover was evaluated in one room-temperature 4 h run after both stations had reached timing lock. One station remained GPS locked as the reference, while the second station’s antenna was disconnected and its local PPS difference was measured. As summarized in Table 9, the difference increased from 0.37 μs at loss onset to 33.10 μs at 240 min. The net drift was 32.73 μs, corresponding to an endpoint-average drift of approximately 8.18 μs/h and a fractional frequency offset of approximately 2.27 ppb. After reconnecting the antenna, GPS was reacquired in 36 s and the measured PPS difference after relock was 0.33 μs.

### 4.6. Field Deployment Test

**Figure 9:**
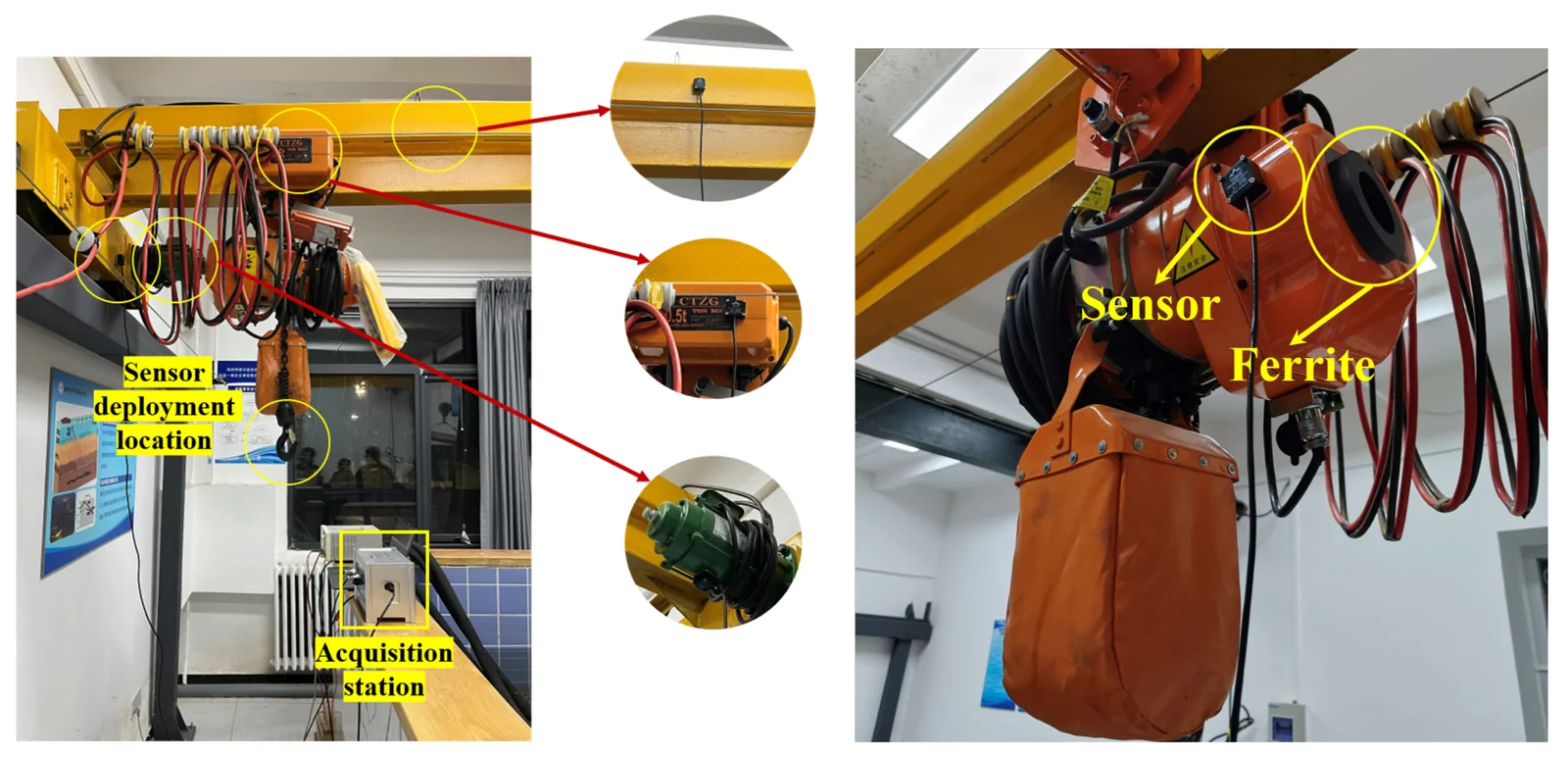
Field deployment and artificial-disturbance setup on the crane-based rig analogue.

Field tests are conducted on a crane-based rig analogue, as shown in Figure 9, to evaluate deployment feasibility, continuous operation, vibration-data availability, and the complete acquisition–transmission–analysis–display workflow under realistic engineering conditions.

Although a crane differs from a deep drilling rig in its overall structure and operating task, the two systems share several relevant mechanical characteristics, including hoisting motion, power transmission, load variation, and coupled structural vibration. Six typical sensor positions are selected according to vibration-transfer paths and expected response characteristics. One station and three triaxial sensors are used; the sensors are moved between two measurement batches to cover the six positions, so the six positions are not recorded simultaneously. After the acquisition station is powered on, GPS synchronization and device initialization are completed, and the system enters continuous acquisition mode at 16 kS/s per channel. At this sampling rate, the ADS1274 digital-filter passband extends to approximately 7.25 kHz ($0.453f_{\mathrm{DATA}}$), which is taken as the usable bandwidth of the reported field measurements. During acquisition, the main control board receives, buffers, stores, and uploads multichannel vibration data, while the host computer displays waveforms, monitors device state, and runs post-acquisition analysis.

**Figure 10:**
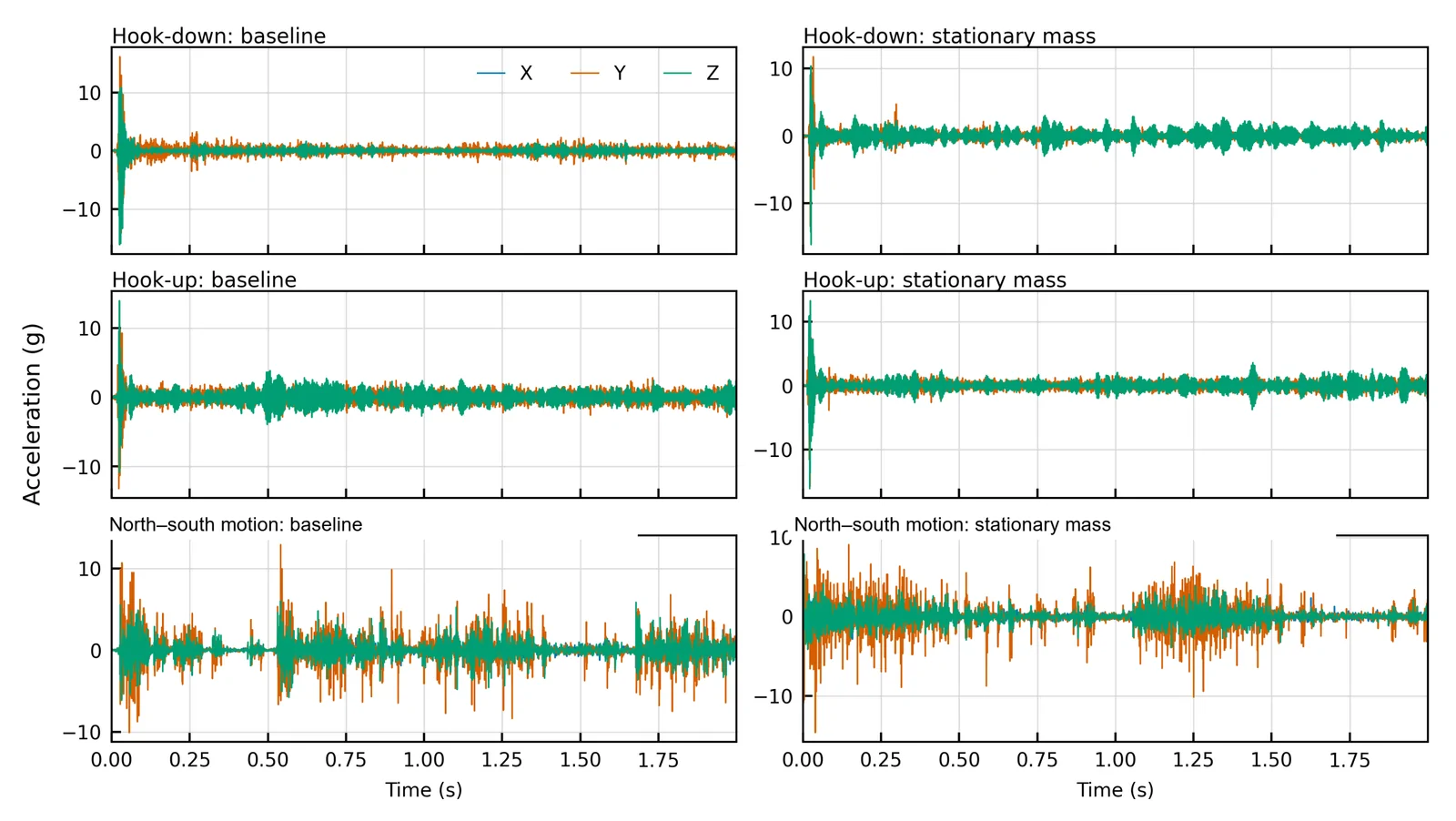
Representative triaxial acceleration waveforms at a 16 kHz sampling rate. The horizontal axis is time in seconds and the vertical axis is acceleration in g; “stationary mass” denotes the removable ferrite attached to the stationary bearing housing.

Several typical operating events are recorded, including east–west movement, north–south movement, steady operation, hook-up motion, and hook-down motion. To introduce a repeatable artificial perturbation, a removable ferrite block is attached to the stationary bearing housing near the motor, as shown in Figure 9. Added masses or counterweights have been used to produce controlled imbalance in rotating-shaft studies [43,44]. The present stationary arrangement is used as a local mass-loading disturbance and differs physically from rotating added-mass configurations, drill-string stick–slip, and whirl. The measured time-domain waveforms are converted from voltage to acceleration using the 156.25 mV/g sensitivity and are shown over a 2 s interval in Figure 10. The records demonstrate that the station captures changes associated with the controlled operating events and local disturbance.

**Figure 11:**
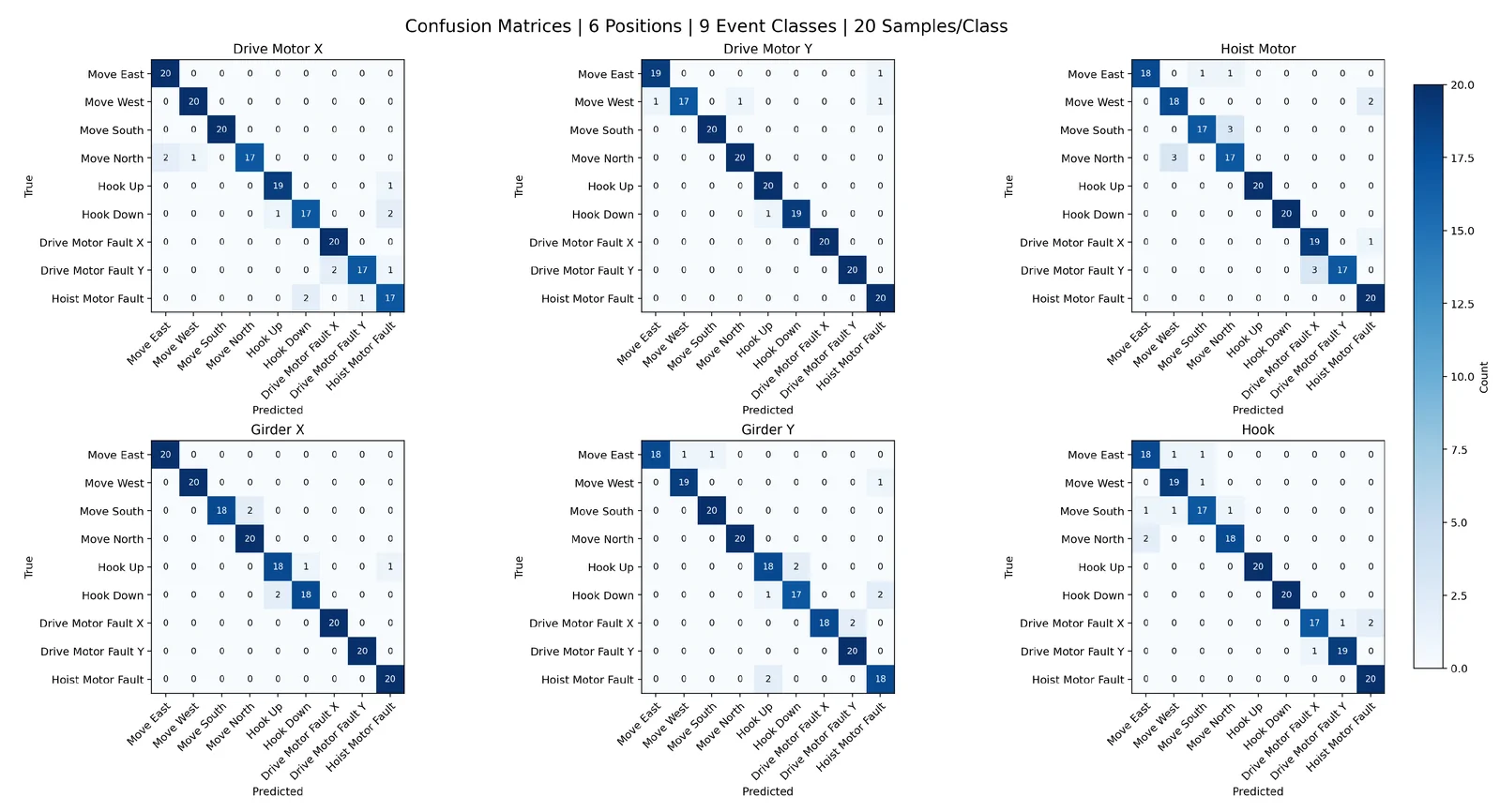
Confusion matrices for six sensor positions and nine predefined classes comprising crane operating events and controlled stationary mass-loading states.

The analysis module is then used to classify the predefined crane operating events and controlled mass-loading states. Independent crane recording runs are divided into training, validation, and test sets at a ratio of 70:15:15 before windowing; windows from one original record are therefore kept within one subset. The confusion matrices in Figure 11 provide a qualitative verification that the predefined states are separable under this protocol using the developed acquisition and software platform.

The confusion-matrix patterns also suggest that the sensitivity of different sensor positions varies with event type. Sensors close to local excitation sources capture clearer vibration responses, whereas events transmitted indirectly through structural coupling are more difficult to separate. This observation indicates that sensor placement should be optimized in future deployment on actual deep drilling rigs.

## 5. Conclusions

This paper presents a Zynq-based triaxial vibration sensing and condition-monitoring station intended for future deep drilling rig applications. A modular acquisition station is developed using an acquisition board, main control board, isolation board, and interface board. The system supports conditioned-voltage triaxial accelerometers, low-noise signal conditioning, 24-bit simultaneous sampling, GPS + OCXO timing, local SSD storage, and wired/wireless communication. A Zynq MPSoC platform is used to combine deterministic PL-side acquisition and timing tasks with PS-side embedded Linux management, storage, transmission, and human–computer interaction.

An intelligent condition-monitoring method based on learnable multichannel weighted fusion and training-distribution-based quantile anomaly detection is also proposed. The method accounts for different anomaly-sensitive responses among vibration axes and estimates a fixed threshold from the reconstruction-error distribution of healthy training samples. Benchmark experiments on a public triaxial bearing dataset show that the proposed method achieves an accuracy of 0.9705 and an F1-score of 0.9704 under the reported protocol. Ablation results further show the contributions of the weighted-fusion and auxiliary-branch design.

Hardware tests demonstrate a mean equivalent input noise of 0.31 μV, a test-derived input-amplitude-to-noise ratio of about 135.08 dB, a gain-linearity residual of 0.935 mV, and a station-to-station local-PPS edge difference of 0.34 μs. A single 4 h room-temperature GPS-loss test gives a net OCXO holdover drift of 32.73 μs, followed by GPS reacquisition in 36 s and a 0.33 μs post-relock PPS difference. A 72 h local-storage run writes 135,895,384,064 bytes with zero recorded DMA, read, write, or process errors. Thermal-stability testing shows that the measured RMS value remains within 354.22–354.25 mV after 30 min of warm-up under a 50 Hz, 1000 mV_pp_ input. The crane-based experiment supports deployment and signal-acquisition feasibility for controlled operating events and a stationary mass-loading disturbance.

Future work will focus on long-term deployment on actual deep drilling rigs, collection of real operational and fault data, optimization of sensor placement, and validation of the proposed monitoring and diagnostic methods under genuine drilling conditions.

## Figures and Tables

**Table 1:** Accelerometer and acquisition-interface specifications used in the field experiment.

Item	Specification
Accelerometer	Customized triaxial voltage-output accelerometer; ±16 g range; −16 g/0 g/+16 g mapped to 0/2.5/5 V; sensitivity 156.25 mV/g; DC–13 kHz bandwidth; noise density <75μg/$\sqrt{\mathrm{Hz}}$
Mounting and supply	Magnetic attachment with screw fixation; 12 VDC supplied by the station
Analog interface	Conditioned 0–5 V input only (no IEPE excitation); 16 kΩ input impedance at 1 kHz; gains 1/10/100/1000
Sampling and filtering	0.5–48 kS/s selectable sampling rate per channel; fixed 10 kHz analog low-pass filter; ADS1274 internal FIR/decimation filter; additional cascaded FPGA FIR filtering at lower sampling rates; 50 Hz digital notch filter

**Table 2:** Comparison with representative baseline implementations on the Triaxial Bearing Vibration Dataset.

Method	Accuracy	Precision	Recall	F1-Score
SVM [34]	0.9430	0.9501	0.9430	0.9435
1D-CNN [16,35]	0.9685	0.9690	0.9685	0.9685
WDCNN [17]	0.9485	0.9538	0.9485	0.9475
AE + threshold [25]	0.9615	0.9615	0.9615	0.9615
Ours	0.9705	0.9720	0.9705	0.9704

**Table 3:** Ablation comparison of the proposed post-acquisition condition-monitoring method.

Method	Description	Accuracy	F1-Score
Ours	Weighted fusion + AE + bypass + threshold	0.9705	0.9704
Fusion-only	Weighted fusion with convolutional classification	0.9485	0.9480
AE + quantile threshold only	Equal-weight fusion + AE + threshold, without auxiliary classification branch	0.9485	0.9415
Fixed-mean fusion CNN	Fixed mean fusion + CNN	0.9495	0.9305
Vanilla 3-channel CNN	Raw three-channel lightweight CNN	0.9615	0.9585

**Table 4:** Functional comparison with commercial vibration acquisition devices.

Metric	Dewesoft IOLITE-8xACC	NI-9232	Self-Developed Station
Channels and sampling	8 channels, simultaneous 40 kS s^−1^ per channel	3 channels, 102.4 kS s^−1^	3 triaxial inputs, 0.5–48 kS s^−1^
Resolution/dynamic range	24 bit, device-specific public spec	24 bit, about 99–105 dBFS	24 bit; 135.08 dB test-derived ratio ^*a*^
Synchronization	PTPv2/EtherCAT options	chassis-dependent	GPS + OCXO
Storage and communication	SD-card options, wired transmission	chassis-dependent, wired transmission	SSD, wired + wireless transmission

The detailed values of commercial devices depend on model configuration and data-rate settings; the table is intended as a functional comparison based on public specifications. ^*a*^ The self-developed value uses Equation (8) and is not directly comparable with a dBFS manufacturer specification.

**Table 5:** Calibration, linearity, and warm-up verification summary.

Item	Procedure	Current Status
Offset calibration	Short input	Mean = 592 nV; std = 22.5 nV; drift range = 550–620 nV over 10 runs.
Gain/linearity	50 Hz sine input, 10–5000 mV_pp_	Mean relative error = –0.080%; maximum absolute relative error = 1.288%; $V_{\mathrm{meas}}=1.000031V_{\mathrm{ref}}+0.236$ mV; $R^{2}=0.99999937$; maximum residual = 0.935 mV.
Thermal warm-up	Room-temperature 50 Hz input	$V_{\mathrm{rms}}$ drift range = 0.35 mV over 240 min; gain-error range = 0.189–0.288%; stable after 30 min.

**Table 6:** Representative thermal-stability results at room temperature (50 Hz, 1000 mV_pp_, sampling rate = 1 kHz, gain = 1).

Time (min)	Equipment Temperature (°C)	$V_{\mathrm{rms},\mathrm{meas}}$ (mV)	Gain Error (%)
0	42.9	354.57	0.288
30	51.2	354.25	0.197
120	50.8	354.23	0.191
240	51.0	354.22	0.189

**Table 7:** Repeatability evaluation results.

Metric	Result	Notes
Offset	mean = 592 nV, std = 22.5 nV, CV = 3.80%	DC mean in 10 short-input runs, drift range = 550–620 nV
Equivalent input noise	mean = 0.31 μV, std = 0.008 μV, CV = 2.58%	10 short-input runs, range = 0.29–0.33 μV
Dynamic range	mean = 135.08 dB	sinusoidal-input saturation test

**Table 8:** Synchronization-accuracy evaluation results.

Pair/Metric	Mean Lag (μs)	Std (μs)	Range (μs)	Notes
Station A–Station B	0.34	0.04	0.32–0.38	local PPS falling edge after GPS + OCXO lock, 10 runs

**Table 9:** Measured PPS difference during one room-temperature GPS-loss holdover test.

Time (min)	0	10	20	30	60	120	180	240
**PPS Difference (μs)**	0.37	1.03	1.93	2.54	5.70	12.90	22.55	33.10

## Data Availability

Field-test data and engineering test records generated for this project cannot be made publicly available due to project requirements of the Deep Earth Probe and Mineral Resources Exploration National Science and Technology Major Project.

## References

[1] Harms U., Koeberl C., Zoback M.D. (2007). Continental Scientific Drilling: A Decade of Progress, and Challenges for the Future.

[2] International Continental Scientific Drilling Program (2026). Scientific Drilling and Deep Earth Exploration. https://www.icdp-online.org.

[3] Leine R.I., van Campen D.H., Keultjes W.J.G. (2002). Stick-slip whirl interaction in drillstring dynamics. J. Vib. Acoust..

[4] Zhu X., Tang L., Yang Q. (2014). A literature review of approaches for stick-slip vibration suppression in oilwell drillstring. Adv. Mech. Eng..

[5] Randall R.B. (2010). Vibration-Based Condition Monitoring.

[6] Jardine A.K.S., Lin D., Banjevic D. (2006). A review on machinery diagnostics and prognostics implementing condition-based maintenance. Mech. Syst. Signal Process..

[7] Lei Y., Li N., Guo L., Li N., Yan T., Lin J. (2018). Machinery health prognostics: A systematic review from data acquisition to RUL prediction. Mech. Syst. Signal Process..

[8] Lei Y., Yang B., Jiang X., Jia F., Li N., Nandi A.K. (2020). Applications of machine learning to machine fault diagnosis: A review and roadmap. Mech. Syst. Signal Process..

[9] Gungor V.C., Hancke G.P. (2009). Industrial wireless sensor networks: Challenges, design principles, and technical approaches. IEEE Trans. Ind. Electron..

[10] Lee J., Bagheri B., Kao H.A. (2015). A cyber-physical systems architecture for Industry 4.0-based manufacturing systems. Manuf. Lett..

[11] Shi W., Cao J., Zhang Q., Li Y., Xu L. (2016). Edge computing: Vision and challenges. IEEE Internet Things J..

[12] Li Z., Fei F., Zhang G. (2022). Edge-to-Cloud IIoT for Condition Monitoring in Manufacturing Systems with Ubiquitous Smart Sensors. Sensors.

[13] Malviya V., Mukherjee I., Tallur S. (2022). Edge-Compatible Convolutional Autoencoder Implemented on FPGA for Anomaly Detection in Vibration Condition-Based Monitoring. IEEE Sens. Lett..

[14] Jia F., Lei Y., Lin J., Zhou X., Lu N. (2016). Deep neural networks: A promising tool for fault characteristic mining and intelligent diagnosis of rotating machinery with massive data. Mech. Syst. Signal Process..

[15] Zhao R., Yan R., Chen Z., Mao K., Wang P., Gao R.X. (2019). Deep learning and its applications to machine health monitoring. Mech. Syst. Signal Process..

[16] Wen L., Li X., Gao L., Zhang Y. (2018). A new convolutional neural network-based data-driven fault diagnosis method. IEEE Trans. Ind. Electron..

[17] Zhang W., Peng G., Li C., Chen Y., Zhang Z. (2017). A new deep learning model for fault diagnosis with good anti-noise and domain adaptation ability on raw vibration signals. Sensors.

[18] Jin Y., Hou L., Chen Y. (2022). A Time Series Transformer based method for the rotating machinery fault diagnosis. Neurocomputing.

[19] Wu K., Nie Y., Wu J., Wang Y. (2023). Prior knowledge-based self-supervised learning for intelligent bearing fault diagnosis with few fault samples. Meas. Sci. Technol..

[20] Kong D., Zhao L., Huang X., Huang W., Ding J., Yao Y., Xu L., Yang P., Yang G. (2023). Self-supervised knowledge mining from unlabeled data for bearing fault diagnosis under limited annotations. Measurement.

[21] El-Wardany T., Gao D., Elbestawi M. (1996). Tool condition monitoring in drilling using vibration signature analysis. Int. J. Mach. Tools Manuf..

[22] Khaleghi B., Khamis A., Karray F.O., Razavi S.N. (2013). Multisensor data fusion: A review of the state-of-the-art. Inf. Fusion.

[23] Li C., Sanchez R.-V., Zurita G., Cerrada M., Cabrera D., Vásquez R.E. (2015). Multimodal deep support vector classification with homologous features and its application to gearbox fault diagnosis. Neurocomputing.

[24] Chandola V., Banerjee A., Kumar V. (2009). Anomaly detection: A survey. ACM Comput. Surv..

[25] Sakurada M., Yairi T. Anomaly detection using autoencoders with nonlinear dimensionality reduction. Proceedings of the MLSDA 2014 2nd Workshop on Machine Learning for Sensory Data Analysis.

[26] Texas Instruments (2011). Quad/Octal, Simultaneous Sampling, 24-Bit Analog-to-Digital Converters Datasheet (Rev. F). Texas Instruments. https://www.ti.com/lit/gpn/ads1274.

[27] AMD Xilinx (2026). Zynq UltraScale+ MPSoC Product Page. https://www.amd.com/en/products/adaptive-socs-and-fpgas/soc/zynq-ultrascale-plus-mpsoc.html.

[28] AMD Xilinx (2025). Zynq UltraScale+ Device Technical Reference Manual, UG1085. AMD Xilinx, 2025. Version 2.5. https://docs.amd.com/v/u/en-US/ug1085-zynq-ultrascale-trm.

[29] Goehringer D. (2014). Reconfigurable multiprocessor systems. ACM Sigarch Comput. Archit. News.

[30] Riley W.J. (2008). Handbook of Frequency Stability Analysis.

[31] Lombardi M.A. (2008). The use of GPS disciplined oscillators as primary frequency standards for calibration and metrology laboratories. NCSLI Meas..

[32] Malhotra P., Vig L., Shroff G., Agarwal P. Long short term memory networks for anomaly detection in time series. Proceedings of the 23rd European Symposium on Artificial Neural Networks, Computational Intelligence and Machine Learning.

[33] Hundman K., Constantinou V., Laporte C., Colwell I., Soderstrom T. Detecting spacecraft anomalies using LSTMs and nonparametric dynamic thresholding. Proceedings of the 24th ACM SIGKDD International Conference on Knowledge Discovery & Data Mining.

[34] Cortes C., Vapnik V. (1995). Support-vector networks. Mach. Learn..

[35] Ince T., Kiranyaz S., Eren L., Askar M., Gabbouj M. (2016). Real-time motor fault detection by 1-D convolutional neural networks. IEEE Trans. Ind. Electron..

[36] Wang J., Wang D., Wang S., Li W., Song K. (2021). Fault diagnosis of bearings based on multi-sensor information fusion and 2D convolutional neural network. IEEE Access.

[37] Xing Z., Liu Y., Wang Q., Li J. (2022). Multi-sensor signals with parallel attention convolutional neural network for bearing fault diagnosis. AIP Adv..

[38] Tong J., Liu C., Zheng J., Pan H. (2023). Multi-sensor information fusion and coordinate attention-based fault diagnosis method and its interpretability research. Eng. Appl. Artif. Intell..

[39] Kumar D., Mehran S., Shaikh M.Z., Hussain M., Chowdhry B.S., Hussain T. (2022). Triaxial bearing vibration dataset of induction motor under varying load conditions. Data Brief.

[40] Triaxial Bearing Vibration Dataset of Induction Motor Under Varying Load Conditions. https://data.mendeley.com/datasets/fm6xzxnf36/2.

[41] (2026). IOLITE Modular Technical Specifications. https://dewesoft.com/products/iolite-modular/tech-specs.

[42] (2026). NI-9232 Specifications. https://www.ni.com/docs/en-US/bundle/ni-9232-specs/page/specs.html.

[43] Wang Q., Hu S., Wang X. (2024). Detection of incipient rotor unbalance fault based on the RIME-VMD and modified-WKN. Sci. Rep..

[44] Wisal M., Oh K.Y. (2023). A New Deep Learning Framework for Imbalance Detection of a Rotating Shaft. Sensors.

